# Supplementary material for: Novel Driver Strength Index highlights important cancer genes in TCGA PanCanAtlas patients
Source: PeerJ. 2022 Aug 11;10:e13860. doi: 10.7717/peerj.13860 (PMC9375969; doi:10.7717/peerj.13860)
Supplement: Supplemental Information 2 [file peerj-10-13860-s002.zip › COHORTS/genes plots/cohorts/2021_8_16_14_9_distribution_cohorts_males_BRCA.pdf]

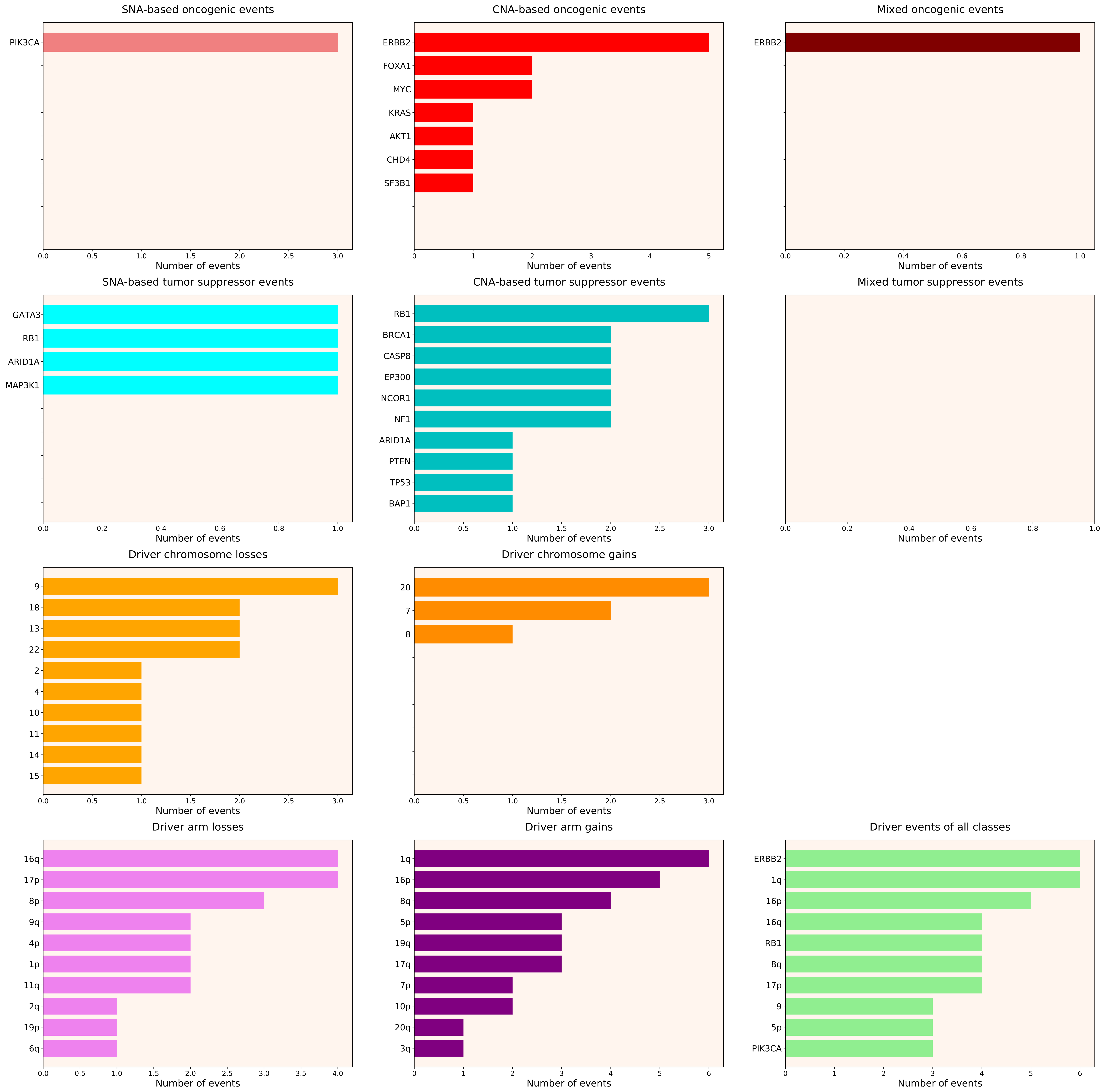
SNA-based oncogenic events
PIK3CA
0.0 0.5 1.0 1.5 2.0 2.5 3.0
Number of events
CNA-based oncogenic events
ERBB2
FOXA1
MYC
KRAS
AKT1
CHD4
SF3B1
0 1 2 3 4 5
Number of events
Mixed oncogenic events
ERBB2
0.0 0.2 0.4 0.6 0.8 1.0
Number of events
SNA-based tumor suppressor events
GATA3
RB1
ARID1A
MAP3K1
0.0 0.2 0.4 0.6 0.8 1.0
Number of events
CNA-based tumor suppressor events
RB1
BRCA1
CASP8
EP300
NCOR1
NF1
ARID1A
PTEN
TP53
BAP1
0.0 0.5 1.0 1.5 2.0 2.5 3.0
Number of events
Mixed tumor suppressor events
0.0 0.2 0.4 0.6 0.8 1.0
Number of events
Driver chromosome losses
9
18
13
22
2
4
10
11
14
15
0.0 0.5 1.0 1.5 2.0 2.5 3.0
Number of events
Driver chromosome gains
20
7
8
0.0 0.5 1.0 1.5 2.0 2.5 3.0
Number of events
Driver arm losses
16q
17p
8p
9q
4p
1p
11q
2q
19p
6q
0.0 0.5 1.0 1.5 2.0 2.5 3.0 3.5 4.0
Number of events
Driver arm gains
1q
16p
8q
5p
19q
17q
7p
10p
20q
3q
0 1 2 3 4 5 6
Number of events
Driver events of all classes
ERBB2
1q
16p
16q
RB1
8q
17p
9
5p
PIK3CA
0 1 2 3 4 5 6
Number of events
